# A Shared Receptor Suggests a Common Ancestry between an Insecticidal *Bacillus thuringiensis* Cry Protein and an Anti-Cancer Parasporin

**DOI:** 10.3390/biom14070795

**Published:** 2024-07-04

**Authors:** Nicole Bryce-Sharron, Mojtaba Nasiri, Tom Powell, Michelle J. West, Neil Crickmore

**Affiliations:** School of Life Sciences, University of Sussex, Brighton BN1 9QG, UK; nb386@sussex.ac.uk (N.B.-S.); mn283@sussex.ac.uk (M.N.); tjp32@sussex.ac.uk (T.P.); m.j.west@sussex.ac.uk (M.J.W.)

**Keywords:** Cry41Aa, Cry1Ca, *Aedes aegypti*, HepG2

## Abstract

Cry toxins, produced by the bacterium *Bacillus thuringiensis*, are of significant agronomic value worldwide due to their potent and highly specific activity against various insect orders. However, some of these pore-forming toxins display specific activity against a range of human cancer cells whilst possessing no known insecticidal activity; Cry41Aa is one such toxin. Cry41Aa has similarities to its insecticidal counterparts in both its 3-domain toxic core structure and pore-forming abilities, but how it has evolved to target human cells is a mystery. This work shows that some insecticidal Cry toxins can enhance the toxicity of Cry41Aa against hepatocellular carcinoma cells, despite possessing no intrinsic toxicity themselves. This interesting crossover is not limited to human cancer cells, as Cry41Aa was found to inhibit some *Aedes*-active Cry toxins in mosquito larval assays. Here, we present findings that suggest that Cry41Aa shares a receptor with several insecticidal toxins, indicating a stronger evolutionary relationship than their divergent activities might suggest.

## 1. Introduction

*Bacillus thuringiensis* (*Bt*) is a Gram-positive, spore-forming bacterium which produces crystalline inclusions during sporulation [1]. These crystalline bodies can house one or more proteins (such as Cry toxins) that possess considerable efficacy and specificity towards invertebrates, which has made these *Bt* Cry toxins valuable to agronomic interests, both as insecticidal agents and as genes expressed in transgenic crops [1]. Cry toxin insect specificity is derived from numerous factors, which include crystal dissolution via compatible pH conditions of the invertebrate midgut, proteolytic cleavage of the protoxin by means of a compatible gut protease, and receptor recognition and binding [1,2].

Cry toxins possess a three-domain toxic core structure, which is highly conserved despite significant divergences in their sequence and target specificity [1]. They all require the same criteria for functionality, namely an environment in which to solubilize the crystal-encased protein, and the proteolytic processing of the N-terminus required for activation [2]. Further still, many known receptors for these Cry toxins are shared across different orders of insects, such as the glycosyl-phosphatidylinositol (GPI) anchored aminopeptidases and alkaline phosphatases [2]. Though the Cry toxins are mostly known and commercialized for their efficacy against insects, a small subgroup of Cry toxins known as the parasporins possess activity against human cancer cells whilst having no known invertebrate targets. Parasporin-3, also known as Cry41Aa, is toxic towards some human cancer cells, which include hepatocellular carcinoma and myeloid leukaemia [3]. We also know that proteolytic activation is required for this activity [4]. Amongst all the specificity determinants between Cry proteins and their targets, receptor interaction is thought to be the most significant step in determining the cytotoxicity of a Cry protein [2]. Multiple receptors have been identified and their interactions with Cry toxins characterized, including some for parasporins [5]. These include Beclin-1 for Parasporin 1 and GPI-anchor protein receptors for Parasporin 2 [6]. The mode of action of a Cry protein may not be limited to one type of receptor; studies have suggested that a toxin might interact with multiple receptors in each insect species and that the interactions between these receptors might enhance the toxic effect [7]. In this work, we have explored the binding characteristics of the Cry41Aa protoxin against hepatocellular carcinoma cells and have demonstrated cross-over between Cry41Aa and some insecticidal Cry toxins, speaking to a closer evolutionary relationship.

## 2. Materials and Methods

### 2.1. Strains, Plasmids, and Constructs

#### 2.1.1. *Bacillus thuringiensis* Strain 4D7

An acrystalliferous strain of *Bacillus thuringiensis* subsp. *kurstaki* was used for the production of all Cry toxins within this work. This strain came from the Bacillus Genetic Stock Center (Ohio State University, OH, USA).

#### 2.1.2. Toxin Expression Plasmids

pHT315 is an *E. coli*/*Bt* shuttle vector containing an origin of replication for both *E. coli* and *Bt*, as well as resistance genes for ampicillin and erythromycin [8]. We have previously used this plasmid as the basis of an expression vector for Cry2Aa and Cry2Ab [9]. In this work, pHTCry41Aa was created by replacing the ORF2 and *cry2A* genes from these constructs with *cry41Aa* and its ORF3 gene. For pHTCry41AaGFP, the non-essential beta-trefoil domain at the C-terminus of Cry41Aa [3] was deleted and GFP was added after the 713th amino acid. To prevent cleavage of the GFP during activation, a unique PreScission protease site (LEVLFQGP) was added after the 40th amino acid, as previously described [4]. In pHTCry41AaHA, an HA tag (YPYDVPDYA) was inserted in pHTCry41Aa after the 825th codon. To create the disabled (DIP) version of Cry41Aa, an A126E mutation was introduced into pHTCry41AaHA. Cry1Ac, Cry1Ca, Cry1Da and Cry4Aa expression plasmids were created by replacing the *cry2Aa* gene from pHTCry2Aa with the alternative coding region. A DIP form of Cry1Ca was created by introducing the mutations N98C and D143C, as identified by Jerga et al. [10]. Finally, Cry11Aa was expressed from a construct in which the gene was cloned in pSVP27 [11].

### 2.2. Growth, Solubilization and Activation of Cry Toxins

*Bacillus thuringiensis* bacteria, containing the Cry toxin genes used in these experiments, were inoculated to flasks containing 1 L half concentration LB broth with the addition of 5 µg/mL erythromycin (chloramphenicol for Cry11Aa expression) and incubated for 3 days at 30 °C, 200 rpm. Several rounds of sonication and centrifugation were undertaken to fully lyse the cells before the spores and crystals were resuspended in ddH_2_O. All Cry toxins were solubilized by pelleting the crude cell lysate, followed by resuspension in 50 mM Na_2_CO_3_ pH 10.5 containing 5 mM dithiothreitol (DTT) and incubation at 37 °C for one hour. Samples were centrifuged at 16,873 g for 2 min, and the supernatant was collected. For the activation of Cry41AaGFP, Pierce HRV 3C protease (1 Unit per 100 µg Thermo Fisher Scientific, Waltham, MA, USA) was used in PreScission reaction buffer and left for 16 h at 4 °C. The enzymatic reaction was stopped by the addition of a Complete mini EDTA-free proteinase inhibitor cocktail solution (Sigma-Aldrich, St. Louis, MO, USA). Cry41Aa and Cry41HADIP were activated by treating the solubilized samples with 1 mg/mL chymotrypsin at 37 °C for one hour. For Cry1Ca and Cry11Aa, trypsin was used instead of chymotrypsin.

### 2.3. Cell Culture

The adherent hepatocellular carcinoma cell line HepG2, purchased from ECACC, was routinely cultured in nunclon surface-treated T-25, T-75, and T-175 flasks (Nunc), using a high glucose DMEM (4.5 g/L, Gibco Life Technologies, Waltham, MA, USA) containing 1% penicillin-streptomycin-L-glutamate (PSG; Gibco Life Technologies) and 10% fetal bovine serum (FCS; Gibco Life Technologies) and incubated at 37 °C with 5% CO_2_ humidified air.

### 2.4. Fluorescence Microscopy Using GFP-Labelled Cry41Aa

HepG2 cells were placed on a sterile microscope cover glass at a density of 2.5 × 10^5^ cells/mL in serum-free DMEM treated with PSG (1%) for 16 h in a 6-well culture plate. Appropriate treatments were added to each well for one hour, the medium was aspirated, and each well washed with PBS. A 4% formaldehyde solution (Sigma-Aldrich) was used to fix the samples. Coverslips were mounted on microscope glass slides using mounting media (plus DAPI), and the edges were sealed. The following parameters were set on the fluorescence microscope (Zeiss LSM880, Oberkochen, Germany): a 64× objective (with immersion oil) was used to obtain a Z-stack scan of cells from bottom to top (cell surface) to obtain micrographs of the whole cell surface. A GFP filter was used to scan the cells for bound GFP-tagged Cry41Aa on the surface of treated cells, whereas a blue filter was used to visualize DAPI staining.

### 2.5. Cellular Competition Assays

Cellular competition assays were carried out in 96-well culture plates (Nunc). Cells were seeded in a volume of 90 µL at a concentration of 2.5 × 10^5^ cells/mL, and allowed to adhere overnight whilst incubated at 37 °C with 5% CO_2_ humidified air. A total of 10 µL of either experimental or control samples were added to wells in triplicate for each condition. For the experimental wells, combinations of Cry41Aa at an EC50 dose of 2 µg/mL and 10–180 fold amounts of competitor were added. Control wells included the application of 10 µL buffer only (50 mM Na_2_CO_3_ pH 10.5), Cry41Aa alone, the competitor toxin at the highest concentration used, or Triton X-100 (0.1%). DMEM was added to additional wells at a volume of 100 µL in triplicate to act as a background fluorescent control. The cells were then incubated for 24 h.

Cell viability was measured the following day using the CellTiter-Blue cell viability assay kit (Promega, Chuo City, Japan). A total of 20 µL of the CellTiter-Blue reagent alamarBlue was added to each well. The plate was incubated for 2 h, before reading using a GloMax-Multi Detection System from Promega. The fluorescence was measured using a green filter with an excitation wavelength of 525 nm and an emission wavelength range of 580–640 nm.

### 2.6. Cellular Binding Assays

Cells were seeded to 6-well culture plates (Nunc), 1 mL per well at a concentration of 1 × 10^6^ cells/mL, and incubated overnight to allow cells to adhere to the plates. Existing DMEM within the 6-well plates was aspirated off, and the toxin/DMEM solutions were added, with a control well receiving only 50 mM Na_2_CO_3_ pH 10.5 buffer. Plates were incubated in the presence of toxin for 1 h. After incubation, DMEM and any unbound toxin were aspirated off, and each well was washed twice in PBS. A total of 70 µL of resuspension buffer (20 mM Tris HCl pH 8 containing 1× phosphatase inhibitors and 1× protease inhibitors) was added to each well, and cells were transferred into the buffer using cell scrapers (Corning, Corning, NY, USA). A total of 70 µL of whole cell lysate was collected from each well and placed in ice-cold Eppendorf tubes to be later analyzed via Western blotting. All binding experiments were repeated at least three times, and the blots contained within this paper are representative of each experimental set.

### 2.7. Western Blotting

Whole cell lysates (10 µL) were combined with 10 µL of 2× SDS-ME (SDS loading buffer + 5% β-mercaptoethanol) and heated at 98 °C for 10 min. These samples were loaded onto a 7.5% SDS PAGE gel at 15 µL per well and ran for 40 min at 200 V. The gel was removed and incubated in semi dry transfer buffer for 5 min while shaking. The protein samples were then transferred to a nitrocellulose membrane (Bio-Rad, Hercules, CA, USA, 0.45 µm) using a Bio-Rad Trans-Blot Semi-Dry Transfer Cell system at 100 mA for 1 h. The nitrocellulose membrane was then equilibrated in 20 mL of 1× PBS buffer for 5 min before incubation for 1 h with 10 mL blocking buffer (1× PBS containing 0.02% tween-20 and 5% dry milk). The blocking buffer was poured off, and the membrane was incubated for a further hour in 5 mL of blocking buffer containing 5 µL of an HRP conjugated anti-HA antibody (Abcam, Cambridge, UK). The membrane was washed in PBS-T 3 times, with a 5 min incubation with each wash. The last wash was poured off, and the membrane was incubated in an enhanced luminol-based chemiluminescent detection (ECL) buffer for 2 min (10 mL of 100 mM Tris HCl pH 8.5, 3 µL H_2_O_2_, 25 µL p-coumaric acid (14.7 mg/mL in DMSO), and 50 µL luminol (88.6 mg/mL in DMSO). The signal was then detected using a UVP ChemStudio imaging system with a 10 min exposure time and displayed within the UVP Life Science VisionWorks software (version 8.20.17096.9551).

For detecting Cry1Ca, the Western blotting process remained the same, save for the use of an in house Cry1C specific primary antibody and a secondary anti-rabbit IgG HRP conjugated antibody (Cell Signalling Technology, Danvers, MA, USA). 

### 2.8. Aedes aegypti Bioassays

*Aedes aegypti* eggs were originally provided by InfraVec2, the adults were fed on defibrinated horse blood and a 10% sucrose solution, and the larvae on goldfish flakes. The insects were kept at 27 °C, 65% humidity. Larvae were selected at the 3rd instar stage of development and placed in 24-well plates, 5 larvae per well and 100 larvae total per condition. Cry1Ca was added at 25 µg/mL, and Cry41Aa was added as a competitor at various concentrations. Water-only and Cry41Aa-only conditions were used as controls. Water in the wells was topped up to a total volume of 3 mL. Larvae were incubated at 27 °C for 24 h, after which the total alive and dead larvae for each condition were recorded.

### 2.9. Data Analysis

All data analysis was carried out using GraphPad Prism version 10.2.2 (Dotmatics, Boston, MA, USA).

## 3. Results

### 3.1. Fluorescence Microscopy Analysis of Binding of the GFP-Tagged Cry41Aa to the Surface of HepG2 Cells

**Figure 1:**
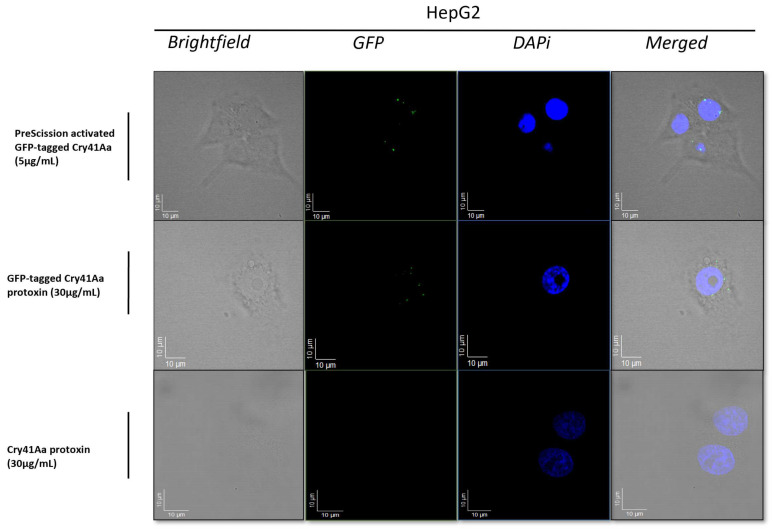
Fluorescent micrographs of HepG2 cells treated with and without Cry41AaGFP. Micrographs show brightfield, GFP, DAPI, and merged (composite) images of each assay using appropriate treatments, and the z-stack level for each image set to the corresponding surface level of cells.

Fluorescence microscopy was carried out to visualize the binding of the Cry41Aa protein to its target cell. The results from the fluorescent micrographs (Figure 1) indicated that Cry41Aa, in both its active and non-toxic protoxin form, could bind to HepG2 cells. Z-stacking focal lengths indicated that the protoxin was localized to the cell surface. This suggested that regardless of proteolytic activation, interaction with the cell membrane still occurred, indicating that N-terminal cleavage has no significant effect on the binding of the protein to its targets.

### 3.2. Immunoblotting Confirms Cry41Aa’s Binding Capability

**Figure 2:**
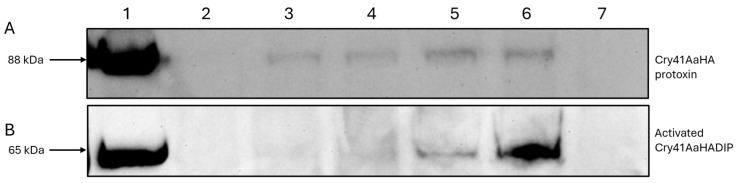
Binding of Cry41AaHA protoxin and activated Cry41AaHADIP toxin to HepG2 cells. (**A**) lane 1, protoxin only; lanes 2–6, whole cell lysates of cells exposed to varying concentrations of Cry41AaHA protoxin (lane 2, 200 µg/mL; lane 3, 300 µg/mL; lane 4, 400 µg/mL; lane 5, 500 µg/mL; lane 6, 600 µg/mL); lane 7, cell only control. (**B**) lane 1, Cry41AaHADIP toxin only; lanes 2–6 show whole cell lysates of cells exposed to varying concentrations of activated Cry41AaHADIP (lane 2, 25 µg/mL; lane 3, 50 µg/mL; lane 4, 100 µg/mL; lane 5, 200 µg/mL; lane 6, 300 µg/mL); lane 7, cell only control.

For many *Bt* Cry toxins, N-terminal proteolytic cleavage has been shown to be a prerequisite for toxicity [9,12]. Though the in vivo activation of Cry41Aa does not occur in the cellular environment, N-terminal cleavage using proteases such as chymotrypsin or proteinase K is nevertheless required for its activation and subsequent activity against cancer cells in vitro [4]. To confirm the fluorescent microscopy analysis which indicated the binding of the protoxin, we compared the signals detected via immunoblotting from conditions where HepG2 cells were exposed to different concentrations of the Cry41AaHA protoxin, and whole cell lysates subjected to immunoblotting. In Figure 2A, lanes 2–6 show a dose-dependent increase in binding for the Cry41Aa protoxin. Lanes 1 and 7 show the HA-tagged protoxin-only and cell-only controls, respectively, suggesting that this signal is specific to the Cry41AaHA protoxin. These results confirm that the protoxin can bind to HepG2 cells despite not undergoing N-terminal cleavage. Binding studies with the activated form of Cry41Aa were complicated by the fact that this form could lyse the target cells. To overcome this, a mutant was created (Cry41AaHADIP), whose mutation (A126E) is thought to prevent pore-formation, rendering the mutant non-toxic. Figure 2B confirms that the activated form of Cry41Aa was also capable of binding in a dose-dependent manner. Compared to the protoxin, however, the binding of the activated toxin could be observed at a lower concentration of toxin.

**Figure 3:**
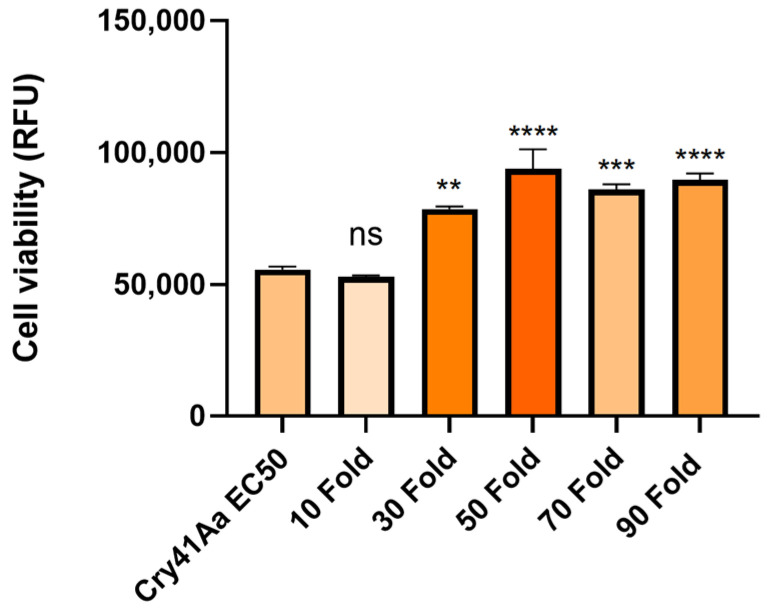
Cell viability of HepG2 cells against activated Cry41Aa, competed with excess Cry41Aa protoxin. Activated Cry41Aa WT administered at an EC50 concentration of 2 µg/mL, competed with excess amounts of Cry41Aa WT protoxin. Cell viability was assessed using the CellTiter-Blue assay. Cell viability was measured in relative fluorescence units (RFU). Error bars show ±SEM. A one-way ANOVA was used to calculate significance. ** *p* = 0.0016, *** *p* = 0.0001, **** *p* ≤ 0.0001, ns = not significant.

A cellular competition assay utilizing HepG2 cells was performed to clarify whether the binding shown in the immunoblotting experiment was specific (receptor-mediated) or nonspecific. For this assay, activated Cry41Aa toxin was competed against increasing amounts of the Cry41Aa protoxin, which possesses no toxicity towards HepG2 cells. As Figure 3 shows, the protoxin form of Cry41Aa has a dose-dependent inhibitory effect on the toxicity of the activated toxin. This indicated that not only is the protoxin form binding to the cells but that it is most likely binding to the same site as the activated form.

### 3.3. Cry41Aa and Insecticidal Cry Toxin Synergism

**Figure 4:**
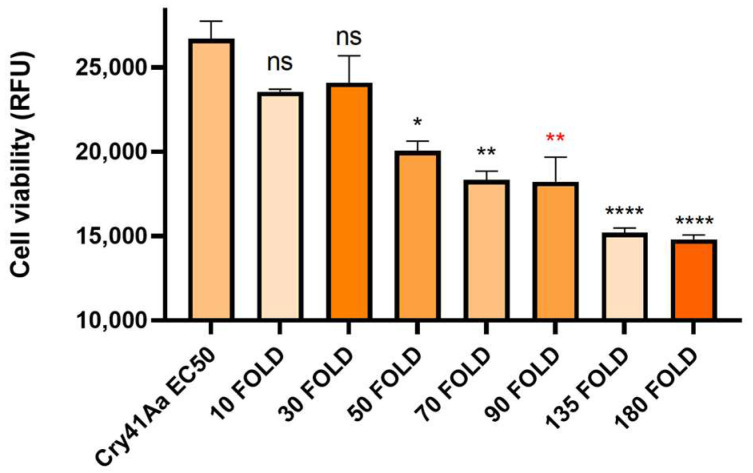
Cell viability of HepG2 cells against activated Cry41Aa, competed with excess activated Cry1Ca. Activated Cry41Aa administered at an EC50 concentration of 2 µg/mL competed with excess amounts of trypsin-activated Cry1Ca. Cell viability was assessed using the CellTiter-Blue assay. Cell viability was measured in relative fluorescence units (RFU). Error bars show ±SEM. A one-way ANOVA was used to calculate significance. * *p* = 0.0166, ** *p* = 0.0020, ** *p* = 0.0018, **** *p* ≤ 0.0001, ns = not significant.

**Figure A1:**
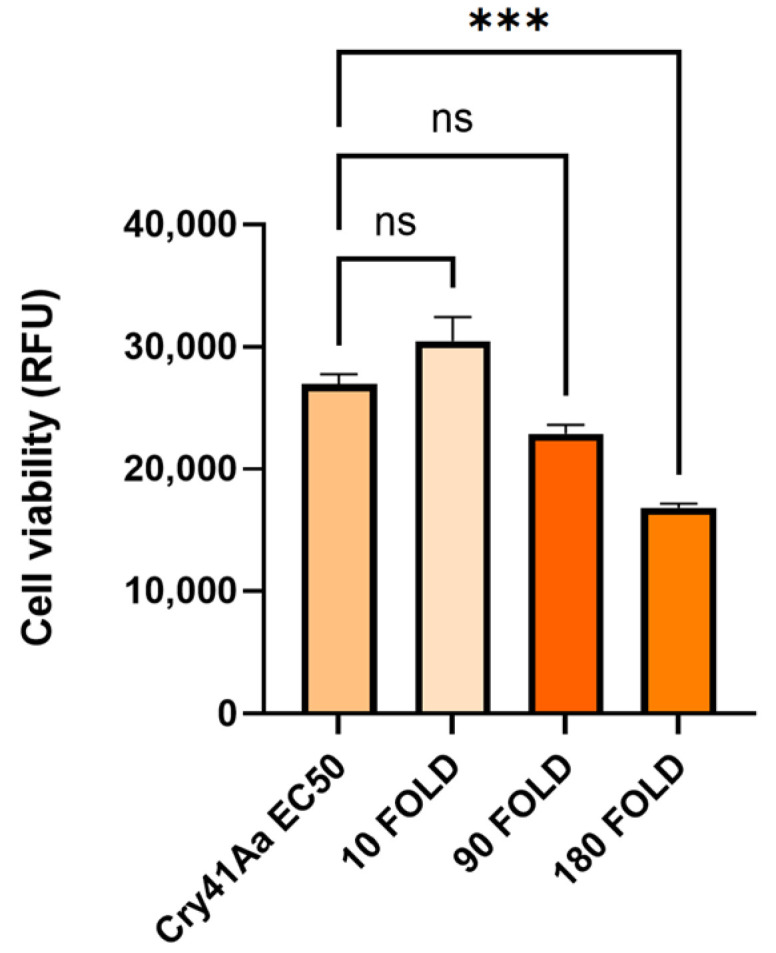
Cell viability of HepG2 cells against activated Cry41Aa, competed with excess activated Cry4Aa. Activated Cry41Aa administered at an EC50 concentration of 2 µg/mL, competed with excess amounts (*w*/*w*) of activated Cry4Aa. Cell viability was assessed using the CellTiter-Blue assay. Cell viability was measured in relative fluorescence units (RFU). Error bars show ±SEM. A one-way ANOVA was used to calculate significance. *** *p* = 0.0006, ns = not significant.

**Figure A2:**
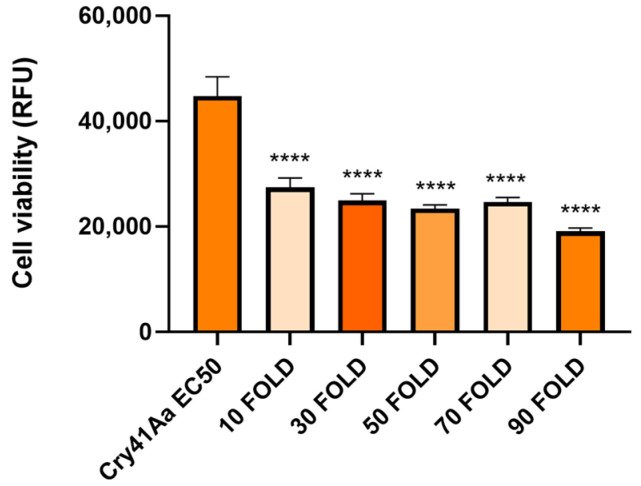
Cell viability of HepG2 cells against activated Cry41Aa, competed with excess activated Cry11Aa. Activated Cry41Aa administered at an EC50 concentration of 2 µg/mL, competed with excess amounts (*w*/*w*) of activated Cry11Aa. Cell viability was assessed using the CellTiter-Blue assay. Cell viability was measured in relative fluorescence units (RFU). Error bars show ±SEM. A one-way ANOVA was used to calculate significance. **** *p* ≤ 0.0001.

Following the inhibition seen in competition with the Cry41Aa protoxin, we investigated whether other Cry toxins could exert any effect on the toxicity of Cry41Aa. To test this, Cry41Aa was competed against increasing amounts of activated Cry1Ca, an insecticidal Cry toxin with efficacy against both lepidopteran and dipteran targets but possessing no toxicity towards HepG2 cells [3]. Unlike the inhibition seen with Cry41Aa and its protoxin form, synergism was observed between Cry41Aa and Cry1Ca, with the combination of the two toxins exerting an increased toxicity compared to Cry41Aa alone. It was also observed that this increase in toxicity correlated with increasing amounts of Cry1Ca (Figure 4). This synergistic effect was not unique to Cry1Ca; two other toxins, Cry11Aa and Cry4Aa, showed similar results, whereas Cry1Da, Cry1Ac, Cry2Aa, and Cry2Ab, had no effect on Cry41Aa toxicity (Table 1, Figure A1 and Figure A2 in Appendix A). None of these insecticidal toxins had any direct effect on HepG2 themselves.

**Figure 5:**
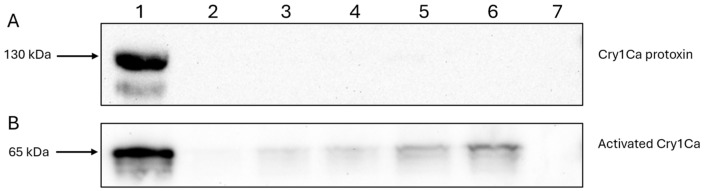
Detection of bound Cry1Ca protoxin and active toxin to HepG2 cells. Lane 1, toxin-only; lanes 2–6 show whole cell lysates of cells exposed to varying concentrations of either Cry1Ca protoxin (**A**) or trypsin-activated Cry1Ca toxin. (**B**) Lane 2, 200 µg/mL; lane 3, 300 µg/mL; lane 4, 400 µg/mL; lane 5, 500 µg/mL; lane 6, 600 µg/mL, lane 7, cell only control.

To determine if Cry1Ca was capable of binding to HepG2, cells were exposed to varying amounts of activated Cry1Ca, before whole cell lysates were subjected to immunoblotting utilizing an anti-Cry1C antibody. As Figure 5B shows, activated Cry1Ca bound HepG2 cells in a dose-dependent manner.

**Figure 6:**
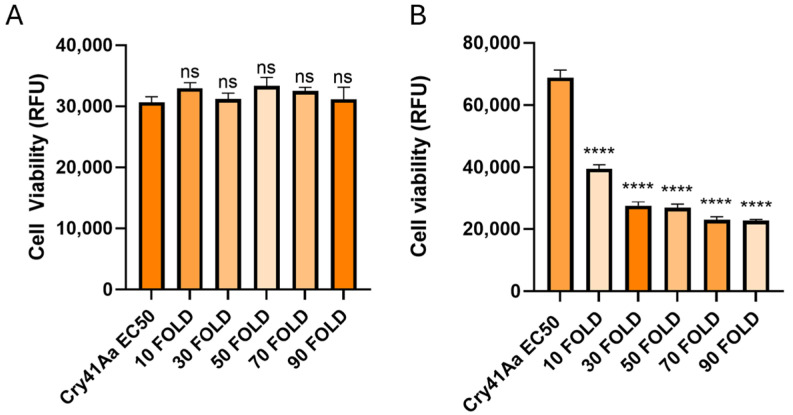
Cell viability of HepG2 cells when treated with activated Cry41Aa and an excess of Cry1Ca protoxin/Cry1CaDIP. Activated Cry41Aa administered at an EC50 concentration of 2 µg/mL, competed with excess amounts of Cry1Ca protoxin (**A**) or activated Cry1CaDIP (**B**). Cell viability was assessed using the CellTiter-Blue assay. Cell viability was measured in relative fluorescence units (RFU). Error bars show ±SEM. A one-way ANOVA was used to calculate significance. ns = not significant, **** *p* ≤ 0.0001.

In contrast, when the same experiment was carried out using the Cry1Ca protoxin, no binding was observed at the concentrations used (Figure 5A). This result is consistent with previous findings, that some insecticidal Cry toxins cannot efficiently bind to their intended targets without first undergoing N-terminal cleavage [12]. When the Cry1Ca protoxin was competed against Cry41Aa, it failed to produce the same synergistic effects as seen with the trypsin-activated Cry1Ca (Figure 6A).

To further test whether Cry1Ca’s synergistic effect on Cry41Aa was due to binding competition, the above assay was repeated with a DIP mutant of Cry1Ca, which is able to bind but not to oligomerize or form pores [10]. As shown in Figure 6B, the activated form of the DIP variant was capable of eliciting a similar synergistic response as the wild type, suggesting that the synergistic effect was directly related to binding.

**Figure 7:**
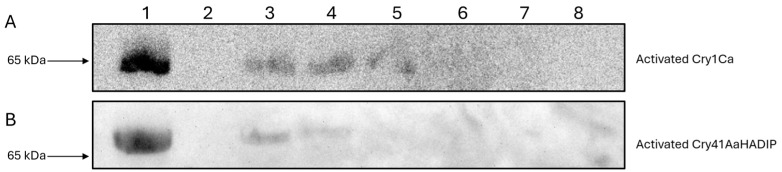
Competition binding assays between Cry41Aa and Cry1Ca. Activated Cry1Ca (**A**) and activated Cry41AaHADIP (**B**), competed against excess unlabeled Cry41Aa protoxin. Lane 1, Cry1Ca/Cry41AaHADIP only; lane 2, unlabeled competitor (Cry41Aa protoxin); lane 3, whole cell lysate exposed to Cry1Ca/Cry41AaHADIP, no competitor; lanes 4–7, Cry1Ca/Cry41AaHADIP, competed against unlabeled competitor at 1:1, 1:5, 1:10, and 1:20 fold excesses, respectively; lane 8, cell only control.

As a further confirmation that activated Cry1Ca, activated Cry41Aa and Cry41 protoxin bind to the same receptor, competition binding studies were undertaken. As Figure 7 shows, Cry41Aa protoxin was able to compete with both activated Cry41Aa and activated Cry1Ca, supporting the idea of a shared receptor.

### 3.4. Cry41Aa Inhibits Cry1Ca Activity in Aedes aegypti Larvae

**Figure 8:**
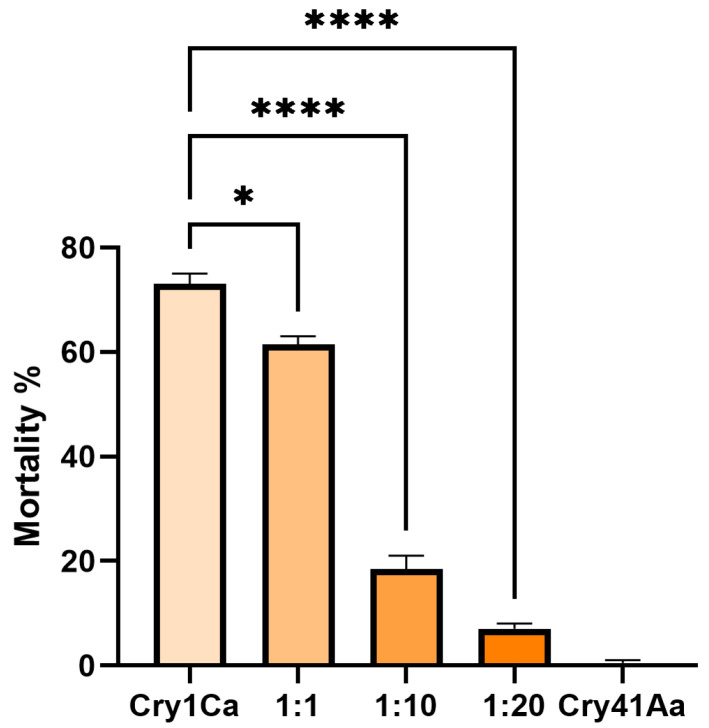
Competition assay between Cry1Ca and excess Cry41Aa, utilizing 3rd instar *Aedes aegypti* larvae. Cry1Ca presented at 25 µg/mL, competed with excess amounts (*w*/*w*) of Cry41Aa. Error bars show ±SEM. A one-way ANOVA was used to calculate significance. * *p* = 0.0130, **** *p* ≤ 0.0001.

**Figure A3:**
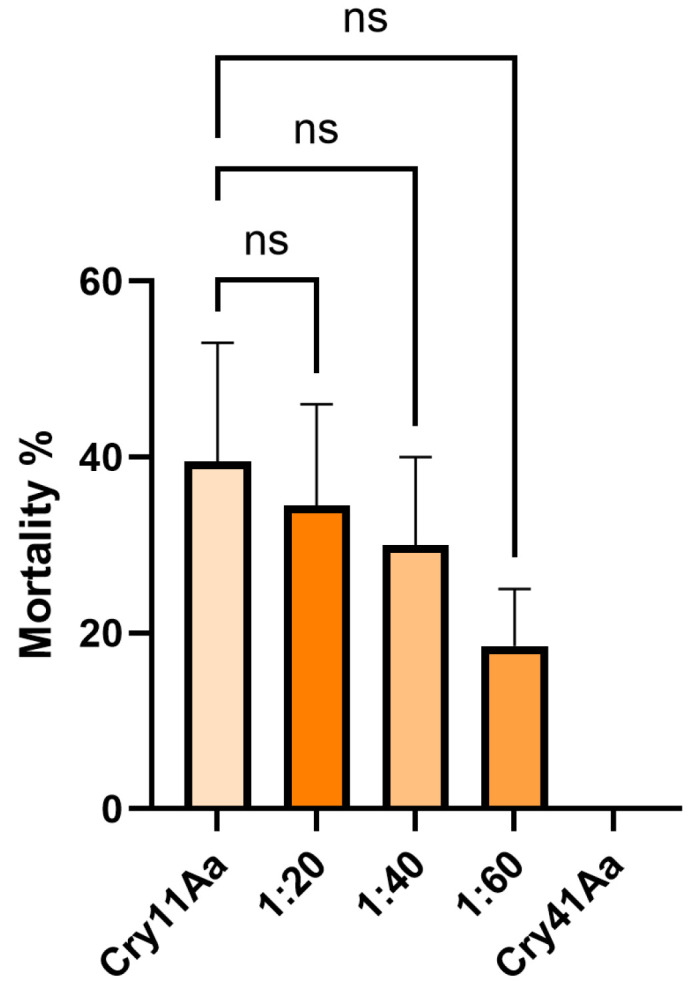
Competition assay between Cry11Aa and excess Cry41Aa, utilizing 3rd instar *Aedes aegypti* larvae. *Aedes*-active Cry11Aa presented at 0.8 µg/mL, competed with excess amounts (*w*/*w*) of Cry41Aa. Error bars show ±SEM. A one-way ANOVA was used to calculate significance. ns = not significant.

Due to Cry1Ca showing a synergistic effect against HepG2 cells with Cry41Aa, we investigated whether a similar effect might occur in a competition assay utilizing an insect system. *Aedes aegypti* mosquitoes were chosen, as they are known to be susceptible to Cry1Ca [13]. In these assays, Cry1Ca was administered at a dosage of 25 µg/mL, along with increasing concentrations of Cry41Aa to 3rd instar *Aedes aegypti* larvae. Unlike the competition assays featuring HepG2 cells, the competitor inhibited rather than synergized. As Figure 8 shows, larval mortality dropped with increasing concentrations of Cry41Aa, indicating that this anti-cancer toxin can inhibit the effects of Cry1Ca through competition. This inhibition was not unique to Cry1Ca, as Cry41Aa was also capable of inhibiting another *Aedes*-active Cry toxin, Cry11Aa, in a similar manner (Figure A3 in Appendix A).

## 4. Discussion

N-terminal activation is believed to be an important step in the mode of action of Cry toxins [12]. Cross-linking studies, using disulfide bonds, have shown that the activation of Cry toxins through the cleavage of the N-terminal peptide segment results in a conformational change, leading to domain I moving away from domains II and III, the insertion of the helical bundle into the membrane, and pore formation [14,15]. Bravo et al., 2007, postulated that this conformational change may expose a region within domain II responsible for toxin–receptor interaction [16]. Our work shows that N-terminal cleavage of Cry41Aa is not required for the toxin to bind to HepG2 cells. It is thought that Cry41Aa, like other Cry toxins, still requires proteolytic processing to insert and form a pore within the membrane, as evidenced by the fact the protoxin is not toxic towards cells [3]. The binding of the Cry41Aa protoxin was confirmed through both fluorescent microscopy and immunoblotting, and it was found to compete with the activated toxin, indicating shared binding sites. Though the Cry41Aa protoxin has been proven to be non-toxic towards HepG2 cells even at relatively high concentrations, a lack of toxicity at any concentration cannot be ruled out [3]. Tabashnik et al., 2015, discussed a dual mode of action hypothesis following findings that Cry1Ab and Cry1Ac protoxins were more potent than the activated toxins in insects with resistance to these toxins, indicating that the mechanisms of resistance can highlight alternative pathways for protoxins to exert toxicity [17].

This study also found that some insecticidal toxins could exert a synergistic effect with Cry41Aa against HepG2 cells, despite these insecticidal toxins not possessing toxicity in their own right. The synergistic effects of *Bt* Cry toxins have been reported in studies since the 1980s [18], and can be the result of several different mechanisms in which combinations of two or more Cry toxins can exert a greater than expected influence [19,20]. The formation of hetero-oligomers between closely related Cry toxins has been shown to confer advantages in receptor binding and subsequent toxicity over monomers and homo-oligomers [21], and thus result in synergism. Toxins may even serve as membrane binding targets for other toxins, as Cyt1A was shown to serve as a functional, membrane-bound receptor for Cry11Aa in *Aedes aegypti* [22].

Of the toxins tested within this study, several were found capable of exerting a synergistic effect with Cry41Aa. Interestingly, all of those with this activity also have activity against *Aedes aegypti*, although it is currently unclear if there is any significance to this observation. Among these, Cry1Ca was shown to bind HepG2 cells in its active, but not protoxin, form. Despite its ability to bind, the exposure of HepG2 cells to activated Cry1Ca, or any of the other insecticidal Cry toxins used, failed to illicit any cell swelling, a characteristic early cellular response of HepG2 cells to Cry41Aa exposure [3]. This absence of cellular response suggests that, whilst possessing the ability to bind a putative receptor is certainly a prerequisite for Cry toxin specificity, other factors such as the orientation of binding or the ability to insert into the host membrane also affect toxicity. This study found that the synergism between Cry41Aa and Cry1Ca was also observed when utilizing a DIP variant of Cry1Ca, indicating that Cry1Ca’s ability to oligomerize had no impact on the synergism observed, likely ruling out the possibility of hetero-oligomer formation as the reason for the synergism. The various interactions between Cry1Ca and Cry41Aa prompted the development of a two-receptor hypothesis. We hypothesize that two receptors exist for Cry41Aa in HepG2 cells, and that one is less effective than the other. Cry41Aa can bind both targets, whilst Cry1Ca, Cry11Aa, and Cry4Aa Cry toxins can only bind to one, the hypothesized “less effective” target. In doing so, these Cry toxins facilitate a synergistic effect by blocking binding to the less effective site, thereby allowing more of the Cry41Aa to bind to the “more effective” target. The observable result is a decrease in cell viability beyond that observed in controls featuring Cry41Aa alone, where more of the toxin has bound to the less or non-effective receptor.

The synergism between the Cry toxins and Cry41Aa in HepG2 cells differs from the effect observed in *Aedes aegypti* larval competition studies. Competition studies have long been a means by which shared receptors and cross-resistance between different Cry toxins have been explored in insect systems [23]. We observed that Cry41Aa could inhibit the toxicity of Cry1Ca and Cry11Aa in larval assays, which suggests that Cry41Aa can bind the same receptor in the *Aedes* midgut. Though the putative receptor for Cry1Ca in *Aedes aegypti* is unknown, it has been elucidated that the receptor for Cry11Aa in *Aedes* is a glycosylphosphatidylinositol (GPI) anchored alkaline phosphatase [24]. Therefore, it is possible that an homologous alkaline phosphatase within mammalian cells may represent a shared binding site for Cry1Ca, Cry11Aa, Cry4Aa and Cry41Aa.

Though long considered to be a non-insecticidal and non-hemolytic Cry toxin, there exists the possibility that Cry41Aa does possess some form of insecticidal ability. Still, any insect species in which it may have an effect has yet to be found. Given Cry41Aa’s interesting crossover with the *Aedes*-active toxins detailed in this work, it could be argued that this parasporin may share a close evolutionary history with Cry toxins, and that perhaps another factor, such as changes to the biochemistry of domain I, have imbued this protein with pore-forming capabilities in mammalian cells rather than in insects.

## Figures and Tables

**Table 1:** A list of insecticidal Cry toxins and their effects when presented with Cry41Aa against HepG2 cells.

Insecticidal Cry Toxin	Insecticidal Cry Toxin Excess by Weight	Cry41Aa Concentration	Effect of Combination
Cry1Ca	10–180 fold	2 µg/mL	Synergism
Cry11Aa	10–90 fold	2 µg/mL	Synergism
Cry4Aa	10–180 fold	2 µg/mL	Synergism
Cry1Ac	10–180 fold	2 µg/mL	No effect
Cry1Da	10–180 fold	2 µg/mL	No effect
Cry2Aa	10–90 fold	2 µg/mL	No effect
Cry2Ab	10–90 fold	2 µg/mL	No effect

## Data Availability

All data are included within the article. Raw data requests and further enquiries can be directed to the corresponding author.
